# Trophic Complexity and the Adaptive Value of Damage-Induced Plant Volatiles

**DOI:** 10.1371/journal.pbio.1001437

**Published:** 2012-11-27

**Authors:** Ian Kaplan

**Affiliations:** Department of Entomology, Purdue University, West Lafayette, Indiana, United States of America

## Abstract

Indirect plant defenses are those facilitating the action of carnivores in ridding plants of their herbivorous consumers, as opposed to directly poisoning or repelling them. Of the numerous and diverse indirect defensive strategies employed by plants, inducible volatile production has garnered the most fascination among plant-insect ecologists. These volatile chemicals are emitted in response to feeding by herbivorous arthropods and serve to guide predators and parasitic wasps to their prey. Implicit in virtually all discussions of plant volatile-carnivore interactions is the premise that plants “call for help” to bodyguards that serve to boost plant fitness by limiting herbivore damage. This, by necessity, assumes a three-trophic level food chain where carnivores benefit plants, a theoretical framework that is conceptually tractable and convenient, but poorly depicts the complexity of food-web dynamics occurring in real communities. Recent work suggests that hyperparasitoids, top consumers acting from the fourth trophic level, exploit the same plant volatile cues used by third trophic level carnivores. Further, hyperparasitoids shift their foraging preferences, specifically cueing in to the odor profile of a plant being damaged by a parasitized herbivore that contains their host compared with damage from an unparasitized herbivore. If this outcome is broadly representative of plant-insect food webs at large, it suggests that damage-induced volatiles may not always be beneficial to plants with major implications for the evolution of anti-herbivore defense and manipulating plant traits to improve biological control in agricultural crops.

## Carnivore Attraction to Herbivore-Damaged Plants

Predation and herbivory are the two most commonly studied ecological interactions, in large part because of the sheer abundance and diversity of prey- and plant-feeding animals in nature. Until somewhat recently, however, the functional roles of predators and herbivores were considered to be largely independent of one another. This meant that those investigating the effects of predators on their herbivorous prey could do so with little to no consideration of plants, which were merely viewed as the substrate upon which predator-prey dynamics played out but not actively involved in the process. The past three decades of ecological research into terrestrial plant-animal communities has dramatically transformed this perspective. It is now widely accepted that plant-herbivore-predator, or tri-trophic, interactions represent a fully integrated and functionally interdependent unit in which plants facilitate carnivores [1],[2]. Logically, it behooves a plant to expose their otherwise cryptic consumers to attack by natural enemies, and thus first and third trophic level organisms are seemingly engaged in a mutually beneficial relationship (i.e., the enemy of my enemy is my friend).

Although plant traits modify carnivore function via numerous mechanistic routes (e.g., extrafloral nectaries secrete a sugar-rich dietary supplement, leaf domatia are small hair tufts that house predaceous mites), the lion's share of theoretical and empirical attention has gone toward volatile plant chemicals emitted in response to herbivore feeding damage that attract the enemies of those herbivores, otherwise known as the “call for help” [3]–[5]. Unlike the aforementioned traits, damage-induced volatiles are near-universal in their distribution across plants and widely exploited by foraging carnivores. Further, because most volatiles are imperceptible to the human nose, at least at the trace concentrations released by plants, their “hidden” messages lend an air of intrigue. Decoding messages encrypted within complex odor blends is no small task, however, both in terms of analytical challenges associated with identifying novel compounds and experimental challenges of interpreting how those compounds affect animal behavior.

**Figure 1:**
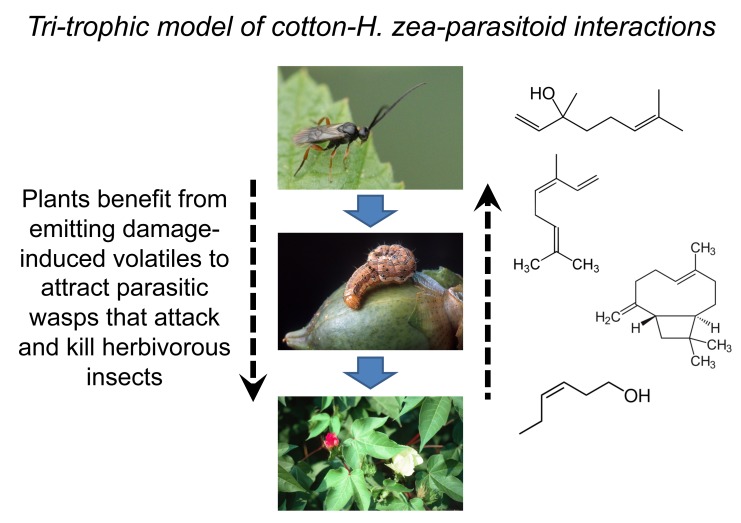
A simple three-trophic level conceptual model based on the well-studied mechanistic linkages between cotton (*Gossypium hirsutum*), the herbivorous insect *Helicoverpa zea*, and the parasitic wasp *Microplitis croceipes*. Solid blue arrows denote who eats whom, whereas dashed black arrows highlight ecological effects spanning non-adjacent trophic levels. Chemical structures represent caterpillar-induced cotton volatiles known to impact parasitoid foraging behavior, e.g., [29],[30]; from top to bottom: linalool, 3,7-dimethyl-1,3,6-octatriene, caryophyllene, and *cis*-3-hexen-1-ol. Photo credits: cotton, Charles T. Bryson, USDA-ARS, Bugwood.org; *H. zea*, Peggy Greb, USDA-ARS, Bugwood.org; *Microplitis* sp., James Lindsey, Ecology of Commanster.

Tri-trophic interactions mediated by plant volatiles are especially well documented for herbivorous insects, mostly caterpillars, because of their small size and thus intimate association with host-plants, which serve as food and housing (Figure 1). Consequently, a foraging carnivore seeking out prey is likely to encounter their victim by simply following the phytochemical trail, resulting in strong selection on plant-feeding insects to engage in stealthy behaviors that evade detection.

## Tri-Trophic Interactions in Light of Food Web Theory

**Figure 2:**
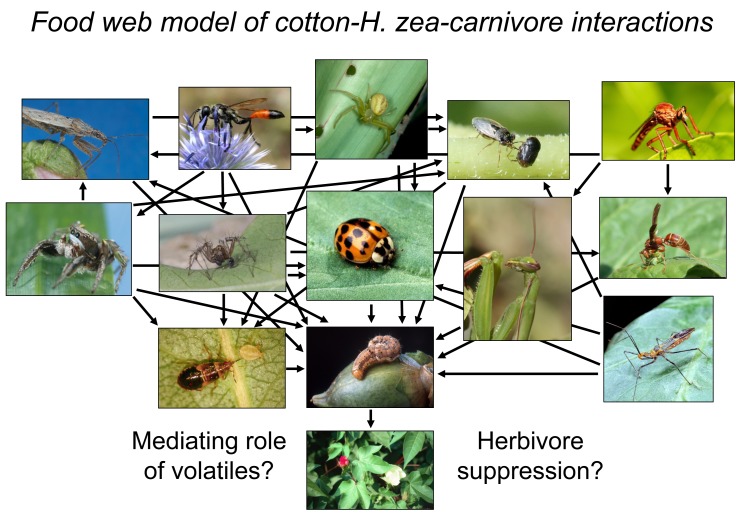
A food web depiction of feeding relationships associated with the caterpillar The trophic diagram is based on direct field observations of predation events by W.H. Whitcomb and K. Bell in Arkansas (US) cotton fields during the 1950s and 1960s, and later reconstructed by [31]. Only a small subset of the carnivore community was included for ease of presentation and the food web thus represents a highly simplified view of trophic dynamics that naturally occur in this system. Photo credits: cotton, Charles T. Bryson, USDA-ARS; *H. zea*, Peggy Greb, USDA-ARS; jumping and lynx spiders and damsel bug, Joseph Berger; crab spider, Frank Peairs, Colorado State University; lady beetle, Scott Bauer, USDA-ARS; big-eyed bug and paper wasp, Russ Ottens, University of Georgia; assassin bug, Clemson University, USDA Cooperative Extension; praying mantis and robber fly, Whitney Cranshaw, Colorado State University; minute pirate bug, Bradley Higbee, Paramount Farming; mud dauber, used with permission from entomart (image available via www.entomart.be). All images (except for the mud dauber) are from Bugwood.org.

The relatively simple picture painted above (herbivore feeds on plant→plant sends out alert signal→carnivores recruit to damaged plant and kill herbivore→plant benefits from reduced herbivory) is the working conceptual model envisioned by virtually all researchers in this field. Is this mechanistic flow chart overly simplistic? Perhaps. The very nature of the term “tri-trophic interactions” is explicitly based on a three-trophic level system where plants, herbivores, and carnivores exist as three discrete groups and carnivores trigger a top-down trophic cascade ( = an indirect positive effect across trophic levels, in this case by suppressing herbivore abundance, thereby releasing plants from consumers). Community ecologists have been debating this vision of trophic dynamics for decades, beginning with Hairston, Smith, and Slobodkin's controversial paper “*Community structure, population control, and competition*” [6]. Intellectual skeptics of this viewpoint, otherwise termed food-web ecologists, take on a more nuanced view, arguing that trophic levels are obscured by pervasive omnivory (feeding across trophic levels) and intraguild predation (predators that eat other predators) [7]–[9]. These terms have barely entered the lexicon of tri-trophic interactions, if at all, but fundamentally alter its core predictions (Figure 2). Moreover, in cases where trophic levels can indeed be discerned, variable food chain length dictates whether or not carnivore impact cascades down to benefit plants; namely, in communities with a distinct fourth trophic level, the beneficial effect of third trophic level consumers is negated [10],[11]. Because of these trophic complexities, the role of carnivores in enhancing plant fitness has been called into serious question [12],[13], particularly in terrestrial ecosystems that tend to form more reticulated food webs than their aquatic counterparts [14]. This further casts a shadow of doubt on the notion that terrestrial plant-insect systems function as linear three-trophic level chains via interactions with volatiles or otherwise.

## Do Plants Benefit from Emitting Carnivore-Attracting Chemicals?

Although it is now recognized that plant volatiles are multi-functional, mediating interactions with pollinators, seed dispersers, neighboring plants, etc. [15], carnivore attraction is still typically assumed to be the primary driver (but see [16]). In simple three-trophic level chains, it is fairly straightforward to envision how carnivores might select for the evolution of volatiles as an inducible plant defense strategy. In complex food webs that are characteristic of real communities, however, the foundation underlying the “call for help” hypothesis becomes more problematic to accept. This is especially so if fourth trophic level organisms eavesdrop on plant cues (presumably) intended for third trophic level consumers, potentially increasing, rather than decreasing, damage to those signaling plants.

A new study by Poelman and colleagues published in this issue of *PLOS Biology* [17] documents this outcome for an assemblage of insects associated with the cruciferous plant *Brassica oleracea*, whose leaves are chewed by larvae of the cabbage white butterfly, *Pieris rapae*. From the third trophic level, two parasitic wasps, *Cotesia rubecula* and *C. glomerata*, lay eggs inside of and eventually kill the herbivorous *P. rapae*. And from the fourth trophic level, another wasp, *Lysibia nana*, parasitizes and kills the two *Cotesia* species (but not *P. rapae*). Wasps that parasitize other parasitic wasps are termed hyperparasitoids, a remarkably diverse and common group but whose ecology and behavior are poorly documented [18]. Through a series of laboratory trials testing wasp odor preferences, chemical analyses of *B. oleracea* volatile profiles, and multi-year field experiments and natural population surveys, Poelman et al. offer strong evidence that hyperparasitoids exploit herbivore-induced plant volatiles in seeking out primary parasitoids as hosts. This finding alone would be a substantial contribution to the existing body of knowledge regarding induced defenses and carnivore attraction in a community context. Two novel insights, however, set this work apart.

First, the authors report an astonishing level of specificity in plant and hyperparasitoid responses to damage by parasitized versus unparasitized caterpillars. Emission of the plant-derived terpenoid (*E*)-DMNT, for example, was 5.6, 7.5, and 15.2, respectively, from the undamaged control, plants damaged by unparasitized caterpillars, and plants damaged by *C. glomerata*-parasitized caterpillars. This means that plant biochemical responses quantitatively differ depending on the parasitism status of the herbivore, in this case the magnitude of (*E*)-DMNT induction was nearly five times greater from plants chewed by parasitized caterpillars. Although induced volatiles are known to differ across herbivore species, e.g., [19], these are among the earliest and best data linking response specificity to intraspecific variation in herbivore condition. Consistent with the volatile data, hyperparasitoids also distinguished between plants on the basis of the status of the inducing herbivore, repeatedly displaying an olfactory preference for plants previously exposed to *C. glomerata*-parasitized (but, interestingly, not *C. rubecula*-parasitized) caterpillars. The authors speculate this putatively adaptive behavior is a consequence of hyperparasitoids realizing higher fitness on the gregarious *C. glomerata* compared with the solitary *C. rubecula*. Overall, these data beg the question—which trophic level is ultimately in the driver's seat? The herbivore or the parasitoid? While the acronym HIPV is often used as shorthand for “herbivore-induced plant volatile,” PIPV, for “parasitoid-induced plant volatile,” may soon enter the vocabulary of plant-insect ecologists!

A second key feature of the Poelman et al. study is its isolation and identification of mediating mechanisms. Parasitic wasps elicit many developmental changes in their caterpillar host, any number of which could be responsible for the above-described specificity patterns. For instance, parasitized herbivores consume less leaf tissue, a behavioral shift that would be expected to impact volatile production given that plant responses to herbivory tend to correlate with damage level. The authors employed an elegant technique whereby caterpillar oral secretions were exogenously applied to a standardized wound on the leaf surface to control for variable tissue damage. Remarkably, hyperparasitoid preference for volatiles of plants attacked by parasitized caterpillars was entirely mediated by salivary chemistry and the effect could be recreated by simply applying saliva from parasitized caterpillars to a leaf wound. Prior work in this [20],[21] and other study systems [22] has revealed the importance of caterpillar oral secretions in modifying plant defense reactions. This example is noteworthy because it integrates salivary-based mechanisms with community-scale ecological outcomes.

## Evolutionary Implications, Agricultural Applications, and Future Directions

Poelman et al.'s work clearly implies selection on fourth trophic level hyperparasitoids to detect subtle shifts in plant volatile constituents and take advantage of this information in host-finding behavior. What is far less clear is whether hyperparasitoids exert reciprocal selection pressure on plants. In the noted study, *Brassica* fitness was not evaluated and, in fact, it is questionable whether the structure of this food web even allows for a top-down cascade of hyperparasitoids on plants. The wasp, *L. nana*, attacks primary parasitoids after they have already killed the herbivore *P. rapae* and emerged from their host's cadaver to spin cocoons and pupate. Thus, hyperparasitism in this system does not necessarily prevent primary parasitoids from protecting plants against caterpillar herbivory, at least on an individual plant basis. That being said, hyperparasitism could reduce wasp abundance at the population-level, making selection pressure on plant chemistry more diffuse and challenging to empirically track. As a whole, studies across plant-insect communities need to begin documenting the plant fitness consequences of variable volatile production and link this relationship with carnivore function, as pleaded for in recent reviews, e.g., [23]. Analogous approaches have proven successful in elucidating the evolution of other putative carnivore-enhancing plant traits such as extrafloral nectaries [24], and early evidence from volatile induction has contributed pieces of this puzzle [25] but not the whole. A central goal should be layering realism onto the existing trophic framework (compare Figures 1 and 2), of which Poelman et al. take a bold step in this direction and set the stage for integrating modern food web ecology into plant volatile-insect interactions.

Beyond the basic evolutionary repercussions of this work, the data also weave together a cautionary tale for manipulating agricultural crop traits to enhance the impact of natural enemies in biological pest control. Increasingly, plant volatiles are eyed as novel tools for augmenting predators and parasitic wasps, but, again, this application is entirely based on the three-trophic level concept [26]. A notable recent field study [27] documented attraction of the lacewing parasitoid, *Anacharis zealandica*, to turnip plots baited with methyl salicylate, the most commonly deployed plant volatile used in biocontrol. Because lacewings are voracious aphid predators, attraction of their parasitoid could indirectly aggravate pest outbreaks from the fourth trophic level. This scenario remains highly speculative, however, until we gain a better understanding of the potential for positive effects of hyperparasitoids and other top consumers on herbivores, which at present is limited [28]. Doing so will require pest management researchers to think more creatively about food web structure in crop environments and the non-target consequences of “calling for help.”
